# Finding Users’ Voice on Social Media: An Investigation of Online Support Groups for Autism-Affected Users on Facebook

**DOI:** 10.3390/ijerph16234804

**Published:** 2019-11-29

**Authors:** Yuehua Zhao, Jin Zhang, Min Wu

**Affiliations:** 1 School of Information Management, Jiangsu Key Laboratory of Data Engineering and Knowledge Service, Nanjing University, Nanjing 210023, China; yuehua@nju.edu.cn; 2 School of Information Studies, University of Wisconsin Milwaukee, Milwaukee, WI 53211, USA; jzhang@uwm.edu; 3 College of Health Sciences, University of Wisconsin Milwaukee, Milwaukee, WI 53211, USA

**Keywords:** consumer health informatics, natural language processing (NLP), online support groups, autism

## Abstract

The trend towards the use of the Internet for health information purposes is rising. Utilization of various forms of social media has been a key interest in consumer health informatics (CHI). To reveal the information needs of autism-affected users, this study centers on the research of users’ interactions and information sharing within autism communities on social media. It aims to understand how autism-affected users utilize support groups on Facebook by applying natural language process (NLP) techniques to unstructured health data in social media. An interactive visualization method (pyLDAvis) was employed to evaluate produced models and visualize the inter-topic distance maps. The revealed topics (e.g., parenting, education, behavior traits) identify issues that individuals with autism were concerned about on a daily basis and how they addressed such concerns in the form of group communication. In addition to general social support, disease-specific information, collective coping strategies, and emotional support were provided as well by group members based on similar personal experiences. This study concluded that Latent Dirichlet Allocation (LDA) is feasible and appropriated to derive topics (focus) from messages posted to the autism support groups on Facebook. The revealed topics help healthcare professionals (content providers) understand autism from users’ perspectives and provide better patient communications.

## 1. Introduction

Today, in the Web 2.0 era, social media are pervasive, rapidly evolving, and increasingly influencing people’s daily life and their health behavior. With the access to information on the social media platforms, people find useful information more effectively and personally than traditional information retrieval through search engines.

According to survey results from parents across the United States, 1 in 40 children (2.5%) have autism spectrum disorder (ASD), representing an estimated 1.5 million children ages 3 to 17 years [1]. Autism is a developmental disorder that appears in the first 3 years of life. It is characterized by substantial deficits in communication and social functioning, as well as restrictive, repetitive, and stereotyped behavior [2]. People with autism experience diverse social and emotional difficulties, such as struggles with social skills and communication impairment [3]. Previous studies have revealed that the special challenges faced by autism patients are associated with social communication, social integration, and social imagination [4,5].

When it comes to adults with autism, the majority of them used social networking sites to seek social connections [3]. Researchers have suggested that social media use appears to be beneficial for individuals with autism in communicating and engaging with others in a comfortable way [3]. Support groups on Facebook provide an efficient platform for autism patients and their caregivers where they can ask for help and advice from other users, make contributions to others, receive assistance from the group members, and share their experiences in the community.

Across different social media platforms, social and informational supports were emphasized as some of the most critical benefits of obtaining health information from online support groups. Through interviews with 14 participants who used support groups on Facebook for weight loss and diabetes management, it was unveiled that participants used Facebook support groups in pursuit of emotional support, motivation, accountability, and advice [6]. Findings from previous studies have identified that informational and emotional supports occupied a significant proportion of the total interaction among users [7].

The topics/focus in social media groups varied time to time. There have been few studies that systematically explore what kind of information is being shared for autism-affected users in social media and how users interact with each other. In this study, data in support groups for autism-affected users on Facebook within a six-month period were collected and analyzed through natural language process (NLP) techniques and the findings were presented by a visualization tool.

The online activities conducted by group members in autism support groups on Facebook produce rich textual content in addition to the interaction connections between users. In this study, the textual content mainly consists of two parts: the original posts submitted by group members, and the comments made by other members. The content created by users in the process of the group interactions reveals the concerns, interests, and other potential information behind the information sharing actions among group members. The content-based analysis uncovers the topics that emerge from group discussions. It provides knowledge regarding the information need of autism-affected users.

The primary research problem of this study is to investigate the content-based characteristics describing the content pattern derived from the communication. The research objects are the autism support groups on Facebook. The autism support groups on Facebook refer to any existing Facebook groups dedicated to autism-related topics. The autism support groups consist of autism patients, their relatives, caregivers, researchers, and physicians. Based on the primary problem, this study aims to address the following research question: *What are the topics that emerged from the discussions and communications posted in autism support groups on Facebook?* The discussions and communications that appeared in the autism support groups were in the form of posts and comments posted by group members.

This study is an investigation of online support groups for autism-affected users on Facebook and the contribution of this study is to find users’ voice on social media, using NLP methods (e.g., Latent Dirichlet Allocation (LDA)) to systematically derive topics (focus) from messages posted to the autism support groups on Facebook. The revealed topics will help healthcare professionals (content providers) understand autism from users’ perspectives and provide better patient communications.

## 2. Materials and Methods

### 2.1. Sampling and Data Collection

#### 2.1.1. Sampling Strategy

The purpose of this study was to investigate the autism support groups on Facebook, thus the screening strategy focused on finding the appropriate Facebook groups. Based on the definition from PubMed Health [8], autism is also called autistic spectrum disorder (ASD) and pervasive developmental disorder (PDD). In order to reach broad data sources, the following autism-related terms were used to search the groups on Facebook: “autism”, “autistic”, “asperger”, “aspie”, and “pervasive developmental disorder”. The search was restricted to Facebook groups. To be included in the study, the groups had to meet the following criteria: (1) the group was related to autism, (2) the group possessed more than 50 group members, and (3) the group operated in English. The first criterion ensured that the sampled groups could be related to the research problem and research questions. The second criterion ensured the conduction of further social network analysis and inferential analysis, while the third criterion ensured the process of content analysis of information shared in the group. For each group identified in the search results, the researcher manually checked the purpose and the operation language of the group through the group title and group description, and recorded the number of group members via group profile. In total, 341 Facebook groups met the requirements.

Through a thorough analysis of the group purposes, all appropriate groups were categorized into ten categories. Table 1 summarizes the categories and the sub-categories of the autism-related Facebook groups.

#### 2.1.2. Group Data Collection Procedure

To achieve sufficient information, the researcher tended to choose the largest and comparative active groups that were available. Five public Facebook autism support groups, each selected from a distinct category (i.e., awareness (under the society and education category), treatment (under the treatment and therapy category), parents (under the care support group category), research (under the commercial and research category), and local support (under the scope category)), became the data sources. According to Facebook, anyone can access the posts in public groups. After joining the groups, a flyer was posted in the groups notifying the process of this study. The comparably largest and most active groups in which group members showed no uncomfortable feelings to the study were selected as the sample groups. In addition, the author intentionally chose groups focusing on diverse topics.

After identifying the sampled groups, data collection for this study centered on the extraction of the interactions and content that appeared in each group. To ensure the fair comparativeness among the groups, a 6-month period was set as the time range of data collection. Data from the sampled public groups on Facebook was collected using NodeXL (The Social Media Research Foundation, Redwood City, USA). NodeXL, produced by Microsoft Research, is an extendible toolkit for network overview, discovery, and exploration [9]. The data import features support data collection from Facebook [9], and export the data into an Excel spreadsheet. To begin the data extraction, we need to go to the “Import” dropdown menu and select “From Facebook Groups”. NodeXL enables the capture of some pertinent aspects of Facebook (e.g., posts, likes, shares, comments). However, due to the limits of Facebook API (Application programming interface), NodeXL can only collect data from the public groups on Facebook.

Data from each of the sampled groups was collected and then saved in Excel spreadsheets for further analysis. On the home page of each group, all of the wall posts created by group members were accessed. Each post included the following information: the Facebook user who created the post, the content of the post, the tag(s) within the post, the post time, the total likes it received, the Facebook user(s) who liked the post, the total share-outs it received, the Facebook user(s) who shared the post, the total number of comments it received, and the content of each comment. For each comment replied to in an original post, it possessed the following components: the Facebook user who made the comment, the content of the comment, the specific time when it was made, the total likes it received, and the Facebook user(s) who liked the comment.

Data from the sampled public groups on Facebook was gathered on December 12–15, 2017. The data collection window was set from April 1, 2017, to September 30, 2017, which covered six months. The time span was flexible to be expanded if there were not sufficient data for data analysis procedures when the final data collection was conducted.

### 2.2. Topic Modeling

#### 2.2.1. Text Preparation Process

Text preparation process refers to a series of steps to prepare the raw text before more in-depth natural language processing, e.g., topic model training. Text preparation commonly consists of the selection, cleansing, and preprocessing of text [10]. In this study, the text preparation process was operated by Python scripts using NLTK toolkits [11].

#### 2.2.2. Latent Dirichlet Allocation (LDA)

Latent dirichlet allocation (LDA) was proposed as a particular generative model for topic discovery [10]. LDA assumes a latent structure consisting of a set of topics, and the words that appear in a paper reflect the particular set of topics [12].

Following the methods introduced by previous researchers, in this study, the LDA model was implemented in the Python with gensim package using the Gibbs sampling inference method [13]. The hyperparameters *α* was set to 50/*K* (*K* is the number of topics) while *β* equals 0.01. The number of iterations was set as 500. The settings of *α* and *β* were based on the suggestion of a previous study where they found it worked well with many different text collections [12].

#### 2.2.3. LDA Model Evaluation

Model evaluation is of importance to the topic modeling methods. The pre-specified numbers of topics influence the performance of the topic model training. The topics discovered by the LDA capture the correlations between words in documents, but the LDA cannot generate the correlations among the captured topics [14]. Too few topics do not allow authors to be distinguished, whereas too many may cause relationships to be weaker [15]. Ideally, topics identified from the documents are supposed to be distinctive from each other. One way to evaluate the LDA model is through the interactive visualization supporting rapid experimentation for interpretive hypotheses [16].

LDAvis handles the model checking problem to aid topic interpretation by displaying the ranking of terms within topics and the relevance among topics [17]. It presents topic–word and topic–topic relationships alongside composition information. In this study, the pyLDAvis package was implemented in Python to visualize the topics generated from the group discussions, as well as assist the modeling checking process. Different values of *K*, or numbers of topics, were tested. The authors assessed the outcomes and decided the most reasonable outcomes based on the data.

**Figure 1:**
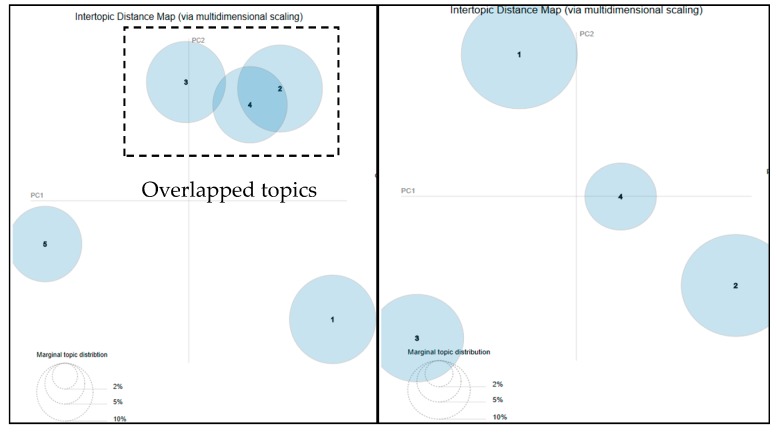
Topic visualization results for different values of K parameter.

To identify the optimal number of topics as the *K* parameter, which is the number of topics in the model training process, interactive visualization methods (pyLDAvis package imported in Python) were employed to evaluate produced models. Using the methods applied in a previous study [18], the number of LDA topics was tuned until it reached a set of non-overlapping clusters that had sufficient distance between each other. The parameter of *K* was given for a series of descending numbers to train the model until the circles representing the topics became separated without any significant overlapping. For example, given the *K* parameter as five, three of the five resulted topics represented as the circles in the inter-topic distance map overlapped with each other (see the left part in Figure 1). When the value of *K* was lowered from five to four, the resulted four topics appeared to be split (see the right part in Figure 1), which meant the generated topics were distinctive to each other. The authors stopped searching for the optimal number of the topics when the circles did not overlap anymore. The size of the circles represents the popularity of the topic within the overall set of topics [18].

All five investigated groups were explored through the model training and evaluation process. After producing the appropriate LDA models, the revealed topics in each group were labeled manually according to the top terms in the topic-term distributions. Labelling topics makes it possible to interpret the corpus to see which concepts are prevalent [19]. The interpretation of a topic can be achieved by examining a ranked list of the most probable terms in that topic [17].

## 3. Results

### 3.1. Description of the Collected Data

As a result, five Facebook autism support groups, each selected from a distinct category, were collected on December 12–15, 2017. All of the group wall posts and group interactions were downloaded by NodeXL. Table 2 presents the basic descriptions of the sampled groups and the collected data. The names of the groups were not revealed for the privacy concerns.

Group 1 is the largest group among the five selected groups, where only 5.1% of the group members participated in the six-month data collection period. Group 3, created for parents, family, and friends of autism patients, had 34.6% of group members engaged in the group discussions. Among the five groups, group 3 had the most group members involved in the group discussions, while group 5 provided the support for people living in a state. After the text preparation process, group 3 remained with the most posts and comments (924 records). In order to protect the user privacy, the Facebook usernames are replaced by serial numbers.

### 3.2. Discussion Topics of Autism Support Groups

#### 3.2.1. Discussion Topics of Group 1 (Awareness Group)

**Figure 2:**
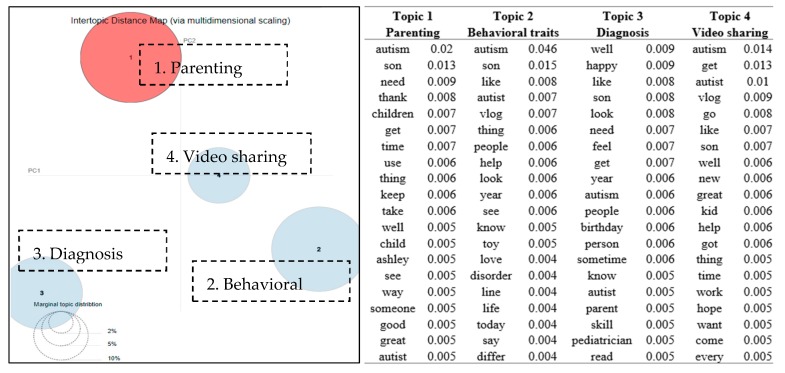
Topic map of four discussion topics in group 1 (awareness group) and top 20 terms and the associated probabilities of the terms to each topic.

The modeling evaluation process indicated that four topics emerged from the posts and comments in group 1. The inter-topic distance map of the four topics (represented by the four circles) is visualized in Figure 2. The right part of Figure 2 lists the top 20 terms and the associated probabilities of the terms to each topic. The four topics thatemerged from the discussions in group 1 were *parenting, behavioral traits, diagnosis,* and *video sharing*. The parenting topic was related to discussions on parents of autistic children sharing their children’s daily life. People shared their children’s accomplishments and sometimes expressed the frustrating issues which happened with their children. Along with talking about the parenting challenges, group members also shared their own behavior traits as patients or their kids’ behaviors. There was a post saying, “*Can anyone tell me if this lining up of toys is a trait of Autism Spectrum Disorder?*” This question raised a number of replies from other group members. Some comments expressed similar observations: “*Our son who has autism LOVES to line up his toys*”. Some stated other opinions: “*Not in of itself. It depends on the age of the child and how the child uses these toys and other toys in other play activities*”. Since autism often appears in early ages of children, parents sometimes struggled with the diagnosis process: “*We keep pushing but the pediatrician just won’t commit to a diagnosis and it’s been 2 1/2 to 3 years now*”. Video sharing topics were contributed mainly by one of the group members, user a41, who posted 30 original messages in the group and was identified as one of the most active users in group 1. After checking with the post content from User a41, he/she has declared himself/herself as a professional speaker with autism. He/she regularly uploaded videos regarding stories about real autism patients and their parents, basic knowledge about autism, how to communicate with people who have autism, school bulling problems for children with autism, etc.

#### 3.2.2. Discussion Topics of Group 2 (Treatment Group)

**Figure 3:**
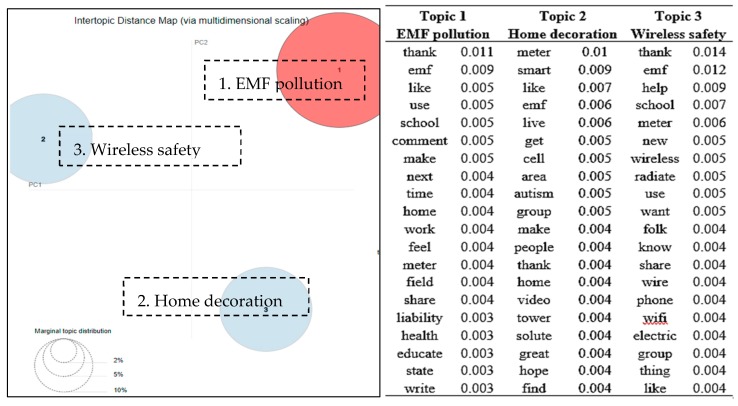
Topic map of three discussion topics in group 2 (treatment group) and top 20 terms and the associated probabilities of the terms to each topic.

Figure 3 gives the global overview of the relationship between the topics based on the established topic-document relationship. Three distinct topics emerged from the discussions in group 2, including *EMF (electromagnetic field) pollution*, *home decoration*, and *wireless safety* (as shown in the right part of Figure 3). The main discussion topic was about the EMF pollution, since the group founder described this group as follows: “*Exploring the emerging link between autism and EMF/wireless, and helping ASD families to heal their children by providing information and resources for reducing their exposure*”. On several occasions, group members shared their advocacy of reducing the EMF pollution. Another discussion theme was regarding home decorations that may reduce or enlarge the EMF (electromagnetic field) pollution. In addition, a number of posts and comments were related to smart meters, including how to install smart meter shields and specific products that can replace the smart meters. Wireless (wifi) networks are one type of sources of EMF according to the National Institute of Environmental Health Sciences [20]. People discussed concerns about a variety of wireless products in the group, such as “*Does anyone still have a child using a Fitbit, Apple watch, or location tracker? Those are all big wireless emitters and can really increase stimming in the kids*”.

#### 3.2.3. Discussion Topics of Group 3 (Parents Group)

**Figure 4:**
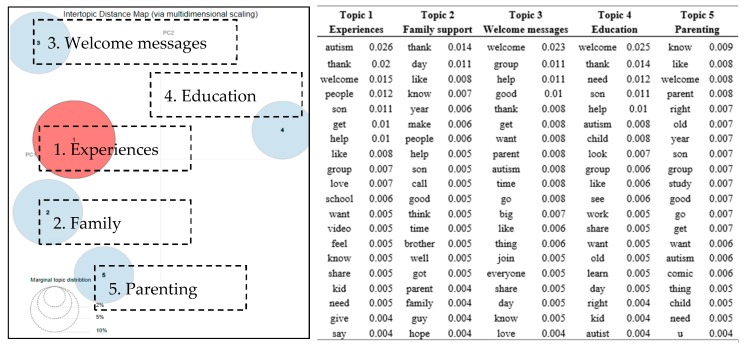
Topic map of five discussion topics in group 3 (parents group) and top 20 terms and the associated probabilities of the terms to each topic.

The five discussion topics drawn from group 3 are shown in Figure 4, while the top terms associated with each topic are listed in the right part of Figure 4. As a group created for parents, *family support,* and *parenting* were not unexpected to be three of the major discussion themes.

Group members shared the stories and experiences about their family members (e.g., brother, son, daughter) in the group, such as “*I was brought to this group because I wanted to connect with people whose lives have been affected by autism. The person in the photo is my baby brother….*”. People also brought up specific questions in being parents of autistic children, such as “*R there any summer camp for my 11-year-old son with autism that in Memphis TN please I need help*”. Another topic, *experiences*, included posts and comments regarding some videos shared by group members. These videos explained the way people on the autism spectrum saw the world and the social difficulties they experienced in real life. With respect to the fourth topic, *education*, people asked questions about how to educate autistic children, such as “*I’m in need of a provider for my 17-year-old girl. …I need someone who can deal with autism. Please help*”. The comments they received provided informational support with methods that might work for their kids, such as “*As a person who has a hyperactive body type, I can emphatically state that heavy work, stretching, and music are all helpful in regulating me*”. In addition to all the discussions regarding specific information needs, both group administrators and other group members posted *welcome messages* (the fifth topic), which showed the welcoming environment of the group to new members.

#### 3.2.4. Discussion Topics of Group 4 (Research Group)

**Figure 5:**
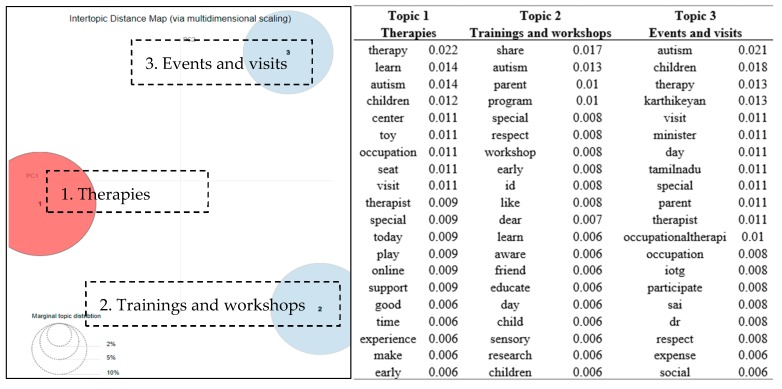
Topic map of three discussion topics in group 4 (research group) and top 20 terms and the associated probabilities of the terms to each topic.

As described on the group main page, group 4 was described as “plays a leading role—locally, nationally and internationally—in developing an improved understanding of the biological and psychosocial basis of autism”. Figure 5 shows that three distinct topics emerged from group 4, while the right part of Figure 5 lists the top 20 terms associated with each topic. The three major topics which appeared in group 4 were related to the *therapies*, the *trainings* and *workshops*, and the *events* and *visits*. Group members talked about various types of therapy for autism patients, such as “*Play therapy builds on the natural way that children learn about themselves and their relationships in the world around them*”. Information regarding trainings and workshops was also shared in the group, such as “*We are excited to announce that our new Online Certification Programme on Play Therapy for Children with Special Needs will launch this August!*” The trainings and workshops discussed in the group were not only for professional therapists but also for parents of autistic kids. User C2 posted most frequently (27 messages) in the group and contributed most to the topic of events and visits. User C2 is the group administrator and serves as an occupational therapist as identified in his/her posts. He/she often updated his professional visits at different places and events he and his colleagues arranged.

#### 3.2.5. Discussion Topics of Group 5 (Local Support Group)

**Figure 6:**
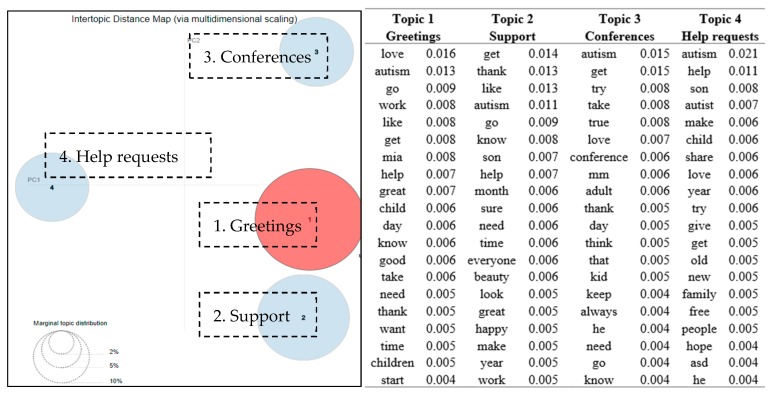
Topic map of four discussion topics in group 5 (Local support group) and top 20 terms and the associated probabilities of the terms to each topic.

The posts and comments in group 5 focused on four topics, as shown in Figure 6. These were *greetings, support, conferences,* and *help requests.* The word “MM” appeared to be among the top words for both the first and the third topic. “MM” is the name of the group founder’s daughter who has autism. A pseudonym was used to protect the privacy of subject. The group founder, user D14, was the most active person in the group, who posted 49 messages and provided 140 comments. He posted photos and daily updates about his daughter. Those posts usually received compliments like “*She is so beautiful love u mia grace!!!*”. As shown in Figure 6, the first two topics were comparatively close to each other. People posted greetings on special days such as on Mother’s Day and someone’s birthday. The third discussion topic was related to an adult autism conference. One of the group members kept posting information regarding the conference, such as the call for presentation flyers, the conference agendas, and the photos of conference presentations. Another discussion topic was questions and answers regarding specific help inquiries, such as “*I am new to this group. I have an amazing 3-year-old son who has recently been given an ASD diagnosis… So I ask, what has been other mom’s or family’s experience with obtaining SSI Disability benefits?*”. Such specific help request tended to receive informational replies from others, such as “*I filed with copy of diagnosis and 4 weeks later start getting payments on child*”.

## 4. Discussion

### 4.1. Social and Informational Exchange in Support Groups on Facebook

Through interviews with 14 participants who used support groups on Facebook for weight loss and diabetes management, it was unveiled that participants used Facebook support groups in pursuit of emotional support, motivation, accountability, and advice [6]. Sugimoto identified informational and emotional supports as the common type of support exchanged in depression online support groups [7]. Sugimoto then summarized findings from previous studies that informational and emotional supports occupied a significant proportion of the total interaction among users [7].

Similar with other support groups on Facebook, social support was also found in the investigated autism support groups. Group members often received comments from others with similar situations and experiences (e.g., “*I can relate entirely and feel this way every day I drop my brave boy at pre-school. Xx*”). Although people may not receive actual information regarding their information needs, the social support they acquired may help with emotional relief.

In addition to general social support, disease-specific information was also exchanged in the support groups on Facebook. Online health communities offer the opportunity for patients to seek and share disease-related information with others who may have similar experiences [21]. A previous study examined online health communities related to arthritis and discovered medication was one of the most popular topics discussed within the community [22]. For individuals in Facebook support groups for presumed ocular histoplasmosis syndrome (POHS), issues regarding diagnosis, treatment, adjustment, and emotional distress were discussed. In the five investigated autism support groups of this study, the following autism-related topics were shared in the Facebook groups: “parenting”, “behavior traits”, “diagnosis”, “home decorations”, “education”, and “therapies”.

As a beneficial result of membership, collective coping strategies were identified as well as timely medical advice based on personal experience, research resources, linkage to services, compassionate support, camaraderie, and social interaction [23]. In this study, it was noticed that group members brought up questions regarding the coping strategies and received multiple suggestions from others with the similar experiences. For example, a mother asked in one of the investigated autism groups: “*what has been other mom’s or family’s experience with obtaining SSI Disability benefits?*”. The post obtained 11 comments including information from others with similar experiences, e.g., “*I filed with copy of diagnosis and 4 weeks later start getting payments on child*”. Another group member replied to the post and expressed the willingness to provide personal help: “*Pm me! I’m happy to help!!!*”. As demonstrated by a previous study, such health communities provide access to experience-based information about particular situations, which many users find more relevant or accessible than information obtained from professionals [6].

Previous studies argued that, unlike face-to-face support groups, instrumental or tangible support was either absent or very rare in online depression support groups [7]. However, it was noticed that group members offered tangible responses when people asked specific questions in the autism support groups in this study. For example, one group member brought up a question: “*I am thinking of purchasing this EMF meter for our home. …Are any of you familiar with this product or have a recommendation for a different meter?*”. Several instrumental and tangible comments replied by others included “*For about the same price, you can get this one: http://www.electricsense.com/10786/cornet-ed88t-emf-meter*/” and “*Tue Cornet is way better for a similar price.*”. In this case, the product recommendations can be considered as informational and useful support for people who did not have such experiences.

Disseminating information with others about upcoming events was identified as one of the most popular things to do on Facebook [24]. In both group 4 (research group) and group 5 (local support group), there was information about a variety of events and conferences shared in the groups. These types of information were also noticed in other Facebook support groups [25]. It suggested that one of the benefits of being involved in support groups on Facebook is to gain access to beneficial information like available events and conferences. It helps group members feel connected and supported, since they could stay current and up-to-date with the groups they choose to be a part of [25].

### 4.2. Emotional Exchange in Facebook Support Groups

The results of this study are consistent with prior studies indicating that that venting is one of the purposes for people who use Facebook groups to fulfill a need to share feelings [26]. Members in online health communities preferred to talk to strangers online about their illness experience than with their offline contacts [27]. In this study, for all of the five investigated autism support groups on Facebook, a prominent theme that conveyed negative emotions can be classified into the venting category (e.g., *“Yes its a very scaring feeling my son is 3 an he takes off on me...My heart sinks”*). It suggested that Facebook groups, similar with other online health communities for long-term conditions [27,28], could serve as not only a place to seek informational and emotional help but also a venue that people could feel free to express the negative feelings.

There are contrasting and possibly conflicting views on the pros and cons to participating in online support communities [26]. In four self-injury groups on Facebook, it was revealed that 3.6% of the total posts were praising or thanking the group [26]. In contrast to the self-injury groups, as shown in Figure 3, Figure 4 and Figure 6, “thank” appeared to be one of the most frequently occurring keywords in three of the five autism support groups. It implied that autism support groups offered a more supportive emotional atmosphere for group members than those of the self-injury groups.

From Figure 2, Figure 3, Figure 4, Figure 5 and Figure 6, many positive words (e.g., “well”, “like”, “thank”, “happy”, “love”, “good”, and “great”) appeared to be the most relevant terms to the discussion topics revealed in the groups. These findings were consistent to a reported healthy and continuous communication loop uncovered in a stutter support group on Facebook [13]. Raj identified that the sense of family which came from the Facebook group helped to diminish feelings of loneliness or isolation for people who had communication barriers [25].

### 4.3. Active Users Lead the Discussions in the Support Groups

As presented above, user A41 in group 1 contributed most in the discussion topic of *video sharing*. User C2 in group 4 shared most of the posts related to the topic of *events and visits*. Part of the posts and comments of the *greetings* topic and the *support* topic were generated by user D14 in group 5. Based on the group interactions, the above three users served as the influential users in their respective group. This suggests that influential users took significant roles in controlling or leading the discussions in their groups.

### 4.4. Limitations

Like most of research studies, there are certain limitations in this study. The first and most obvious one of all the limitations to this study concerns the sampling and data collection. Facebook groups were the only social community on social media addressed in this study. In addition, due to ethical considerations, only public Facebook groups were investigated in this study. Furthermore, the sampled Facebook groups might not be representative of all autism-related support groups on Facebook. Also, this data collection period was six months. The limited time window might be unable to provide a whole picture of the group behaviors.

Another limitation is related to the research methods adopted in this study. This study identified and examined only the quantitative aspect of the autism support groups on Facebook. Understanding the motivations behind the group interactions and group posts could shed insight on the meanings and purposes of the autism-related social communities on social media. However, this study did not interview group members that participated in the support groups on Facebook. The inclusion of interviews or questionnaires was not considered in this study but should be conducted in the future study.

## 5. Conclusions

This study concluded that latent dirichlet allocation (LDA) is feasible and appropriated to derive topics (focus) from messages posted to the autism support groups on Facebook. An interactive visualization method (pyLDAvis) was employed to evaluate produced models and visualize the inter-topic distance maps.

As a result, distinct discussion topics were summarized and labeled in each group. Each group had certain distinctive discussion topics that related to the purposes of the groups. Parenting was a common theme in group 1 and group 3. In addition, several time-sensitive topics appeared during the group discussions. Group members greeted about Mother’s Day during May.

Theoretically, the results of this study align with previous studies that have demonstrated the significance of social media for autism users. The unique implication of this study is to identify autism support groups on Facebook as a source of informational, social, and emotional support for autism-related users. This observation suggested new opportunities of using Facebook to help users who suffer from autism. The findings regarding discussion topics appearing in the autism support groups on Facebook revealed the information needs of autism-related users. In addition, it examined that the informational support, such as specific strategies to deal with autistic kids, was provided in those support groups on Facebook.

This study examined topics derived from messages posted to the autism support groups on Facebook. These topics can also be used as the road map for the design of autism websites and the creation of subject directories for social media information organization. In addition, the revealed topics help professionals understand autism from users’ perspectives. The keywords can be used to assist the thesaurus and subject headings. In addition, the symptom-related content (e.g., lining toys, reading comic books) which emerged from the group discussions aids the screening for parents who wonder whether their children show autism symptoms. The relationships between keywords and topics identified through topic modeling may also be used to build recommendation mechanisms for the Facebook group platform and social question and answer (Q &A) websites.

These focus and overviews of autism-related users in this study can be used to improve the design of autism websites and the creation of subject directories for social media information organization and increases the usage of the digital content. In addition, the revealed topics help healthcare professionals (content providers) understand autism from users’ perspectives and provide better patient communications.

## Figures and Tables

**Table 1:** Categories and sub-categories of the autism-related Facebook groups.

Category	Sub-category	Category	Sub-category
**Autism patient group**	Women Teenager Christian Adults Youth Islam LGBT (Lesbian, Gay, Bisexual, and Transgender)	**Autism with other related diseases**	Sensory Processing Disorder Ehlers Danlos Syndrome/ Hypermobility Syndrome Neurological/behavioral challenge Down Syndrome Type 1 Diabetes Dyslexia
**Care support group**	Mother General family members and caregivers Partner (wife/spouse) Parents	**Society and education**	Awareness Fundraising and charity Art Education Non-profit organization
**Treatment and therapy**	Essential Oils Chlorine dioxide MAPS (Medical Academy of Pediatric Special Needs) Treatment	**Patient and society**	Friend seeking Relationship Job Protest
**Specific autism type**	Severe autism High-functioning autism	**Commercial and research**	Consumer group Consultancy services Research
**Scope**	Local support National support Global support	**Special discussion**	Buying and selling Gift

**Table 2:** Descriptive statistics for the five collected autism support groups.

Group	Category	Members	Involved Members	Posts and Comments
**Group 1**	Awareness	5902	299	314
**Group 2**	Treatment	1577	297	259
**Group 3**	Parents	1513	523	924
**Group 4**	Research	2603	156	88
**Group 5**	Local support	2847	438	756
